# Graphene Oxide/Polyethyleneimine-Modified Cation Exchange Membrane for Efficient Selective Recovery of Ammonia Nitrogen from Wastewater

**DOI:** 10.3390/membranes13080726

**Published:** 2023-08-10

**Authors:** Yuanyuan Yu, Qin Zeng, Haoquan Zhang, Maoqin Ao, Jingmei Yao, Chun Yang, Svetlozar Velizarov, Le Han

**Affiliations:** 1 Key Laboratory of the Three Gorges Reservoir Region’s Eco-Environment, Ministry of Education, College of Environment and Ecology, Chongqing University, Chongqing 400045, China; yuyuanyuanxy@163.com (Y.Y.); zengqin202308@163.com (Q.Z.); zhang_haoquan@foxmail.com (H.Z.); aomaoqin@163.com (M.A.); yaojm@cqu.edu.cn (J.Y.); eric.chun.yang@163.com (C.Y.); 2 LAQV/REQUIMTE, Department of Chemistry, NOVA School of Science and Technology, FCT NOVA, Universidade NOVA de Lisboa, 2829-516 Caparica, Portugal

**Keywords:** cation exchange membrane, graphene oxide, polyethyleneimine, Donnan dialysis, selective NH_4_^+^ separation

## Abstract

Competition for the migration of interfering cations limits the scale-up and implementation of the Donnan dialysis process for the recovery of ammonia nitrogen (NH_4_^+^-N) from wastewater in practice. Highly efficient selective permeation of NH_4_^+^ through a cation exchange membrane (CEM) is expected to be modulated via tuning the surface charge and structure of CEM. In this work, a novel CEM was designed to form a graphene oxide (GO)-polyethyleneimine (PEI) cross-linked layer by introducing self-assembling layers of GO and PEI on the surface of a commercial CEM, which rationally regulates the surface charge and structure of the membrane. The resulting positively charged membrane surface exhibits stronger repulsion for divalent cations compared to monovalent cations according to Coulomb’s law, while, simultaneously, GO forms π–metal cation conjugates between metal cations (e.g., Mg^2+^ and Ca^2+^), thus limiting metal cation transport across the membrane. During the DD process, higher NH_4_^+^ concentrations resulted in a longer time to reach Donnan equilibrium and higher NH_4_^+^ flux, while increased Mg^2+^ concentrations resulted in lower NH_4_^+^ flux (from 0.414 to 0.213 mol·m^−2^·h^−1^). Using the synergistic effect of electrostatic interaction and non-covalent cross-linking, the designed membrane, referred to as GO-PEI (20) and prepared by a 20 min impregnation in the GO-PEI mixture, exhibited an NH_4_^+^ transport rate of 0.429 mol·m^−2^·h^−1^ and a Mg^2+^ transport rate of 0.003 mol·m^−2^·h^−1^ in single-salt solution tests and an NH_4_^+^/Mg^2+^ selectivity of 15.46, outperforming those of the unmodified and PEI membranes (1.30 and 5.74, respectively). In mixed salt solution tests, the GO-PEI (20) membrane showed a selectivity of 15.46 (~1.36, the unmodified membrane) for NH_4_^+^/Mg^2+^ and a good structural stability after 72 h of continuous operation. Therefore, this facile surface charge modulation approach provides a promising avenue for achieving efficient NH_4_^+^-selective separation by modified CEMs.

## 1. Introduction

The recovery of ammonium (e.g., NH_4_^+^-N) from wastewater is one of the key challenges in the process of wastewater resource recovery [1,2,3], since traditional processes for its removal require a relatively high energy consumption and lead to greenhouse gas emissions [4,5,6]. Techniques for enriching target ions via IEMs which are able to selectively transfer oppositely charged ions (counter ions) across the membrane have been widely developed [7], such as electrodialysis (ED) [8,9], bipolar membrane electrodialysis (BMED) [10] and Donnan dialysis (DD) [11]. DD is an electrochemical potential gradient-driven membrane separation technology using ion exchange membranes (IEMs) for the recovery of NH_4_^+^-N at ambient temperature and pressure, with low energy consumption and simple operation. The process achieves reverse concentration gradient transfer of target counter ions without applying an external electric field (i.e., minimal energy demands) and is commonly used for ion enrichment [12,13]. Chen et al. [11] showed that DD can be successfully applied to the enrichment of NH_4_^+^-N in wastewater.

Cation exchange membrane (CEM) performance has a significant impact on the recovery efficiency of NH_4_^+^-N. Multiple ions are often present in the actual effluent and during the ion exchange process, therefore other cations (Na^+^, K^+^, Ca^2+^, Mg^2+^, etc.) can compete with the migration of NH_4_^+^, leading to a lower NH_4_^+^ permeation flux [14]. As a common metal ion in wastewater, Mg^2+^ is characterized by a relatively low positive charge similar to that of NH_4_^+^, which can compete with the transmembrane transport of NH_4_^+^ [15]. The traditional CEM is unable to achieve selective transport of NH_4_^+^, which makes the practical use of DD less valuable. Thus, a dedicated modification of the conventional CEM, combining both high selectivity and permeability of the target ions, is required to achieve efficient recovery of NH_4_^+^ from wastewater. Currently, techniques such as cross-linking, annealing treatment and surface grafting to reduce the pore size and improve the denseness of the membrane can enhance the membrane’s pore sieving effect on high-valent cations [16] but cause a reduction in the permeability of the target ions [17]. Modulation of the hydrophobic properties of the membrane by polymer blending enhances the difference in affinity between different ions and the membrane and enables selective screening of ions [18,19]. Furthermore, the formation of positively charged layers with opposite charge properties on a CEM surface by layer deposition and adsorption techniques to enhance the electrostatic repulsion of high-valent cations by the CEM can effectively improve the selectivity of the membrane for mono/divalent cations [20,21]. Pan et al. [22] showed that polyethyleneimine (PEI) can effectively improve the IEM selectivity of for monovalent/divalent ions. Nevertheless, the above methods may lead to problems, such as reduced membrane ion exchange capacity and increased surface resistance, which prevent selective separation and efficient transport of the target ions simultaneously.

Carbon-based nanomaterials with high electrical conductivity, mechanical strength and corrosion resistance are widely used for membrane modification, such as graphene quantum dots (GQDs) [23], carbon nanotubes (CNTs) [24,25] and graphene oxide (GO) [26]. GO contains many polar groups such as carboxyl groups, hydroxyl groups and epoxy groups on its lamellae, which have favorable hydrophilicity and stability [27]. Through the interactions between functional groups, GO can be chemically modified, which makes it widely considered in the process of membrane material preparation and modification [16,28]. The use of GO as a modifying material to modulate the surface chemistry and internal structure of organic polymer membranes can impart high flux, high selectivity and excellent antifouling properties to the membranes [29]. Ding et al. [30] prepared GO-PEI hybrid membranes by dip-coating and used them in nanofiltration processes with different solvent systems, showing that the introduction of GO can greatly improve the rejection performance, swelling resistance and stability of the membranes. Furthermore, GO as a carbon-based material has certain electrical conductivity, which can reduce the electrical resistance of the modified membrane to a certain extent and thus facilitate the transport of ions across the membrane. Hence, the use of GO and polyelectrolyte coating synergistically to modulate the membrane surface properties and internal structure may achieve efficient selective separation of NH_4_^+^.

In this work, a GO-PEI composite membrane was prepared and extensively investigated in terms of its feasibility to be used in a DD operation for NH_4_^+^ recovery from aqueous solutions. The Donnan percolation phenomenon at the interface of the GO-PEI membrane and the control (unmodified) membrane was studied and evaluated separately in order to allow for a straightforward comparison.

## 2. Materials and Methods

### 2.1. Materials

Neosepta cation exchange (CMX) membrane was purchased from Japan’s Astom Co., Ltd. (Tokyo, Japan) Graphene oxide (GO, JCGO-1-2-W, diameter 0.8–3 μm, thickness 0.8–1.2 nm and single layer ratio > 99.8%) was acquired from Nanjing Jicang Nanotechnology Co., Ltd. (Nanjing, China) Polyethyleneimine (PEI, M_w_ = 70 kDa) and hydrochloric acid (HCl, ≥99.70%) were purchased from Aladdin Co., Ltd. (Seoul, Republic of Korea) Sodium hydroxide (NaOH, ≥99.70%), ammonium chloride (NH_4_Cl, ≥99.70%) and sodium potassium tartrate (C_4_H_4_O_6_KHa·4H_2_O, ≥99.70%) were purchased from Chongqing Chuandong Chemical Co., Ltd. (Chongqing, China) Nascent reagent (Type I), magnesium chloride (MgCl_2_·6H_2_O) and phenolphthalein (C_20_H_24_O_4_) were purchased from Chengdu Kolon Chemical Co., Ltd. (Chengdu, China) Sodium chloride (NaCl, ≥99.70%) was obtained from Chongqing Boyi Chemical Reagent Co., Ltd. (Chongqing, China). All solutions were prepared with Milli-Q water (≥18.2 MΩ cm).

### 2.2. Preparation of GO-PEI Membrane

**Figure 1:**
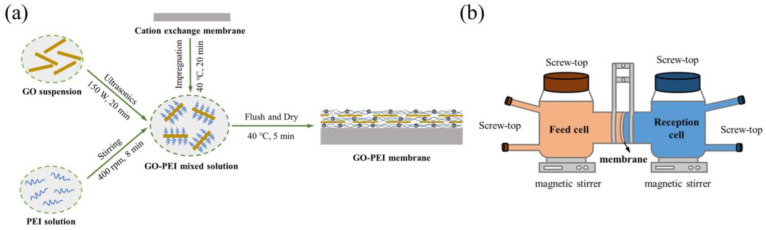
Schematic representation of (**a**) GO-PEI membrane preparation and (**b**) the Donnan dialysis unit.

GO-PEI-modified membranes were prepared using self-assembly (see Figure 1a). Firstly, 0.05 g PEI powder was put into a beaker, dissolved with 100 mL Milli-Q water and stirred for 8 min (400 rpm) to prepare 0.5 g/L PEI solution. Then, 3 mL of GO dispersion (1 mg/mL) after a 20 min ultrasonic-bath treatment (150 W) was added to the above PEI dispersion and stirring was continued at 400 rpm for 2 min to prepare a GO-PEI mixed solution. Subsequently, a cut 6 cm diameter cation exchange membrane was impregnated with the GO-PEI mixture solution with continuous stirring at 40 °C for 20 min to deposit positively charged PEI and negatively charged GO on the membrane surface. After impregnation, the GO-PEI membrane was withdrawn and rinsed with Milli-Q water for 1 min to wash out the residual GO-PEI mixture on the membrane surface. Finally, the GO-PEI membrane was placed in a glass Petri dish and dried in a blast oven at 40 °C for 5 min until the surface was free of moisture and then stored. Impregnated GO-PEI membranes with impregnation times of 0, 5, 15, 20, 30 and 40 min were prepared and named GO-PEI (X) membranes, where X indicates the impregnation time. Furthermore, PEI-modified membranes with impregnation times of 5, 20 and 40 min, named PEI (X), where X represents the impregnation time, were also prepared for comparison according to the method described without adding GO.

### 2.3. Physicochemical Characterizations of Membranes

The surface and cross-sectional structural morphology of the membranes was observed using scanning electron microscopy with energy dispersive X-ray spectroscopy (SEM-EDS, ZEISS Sigma 300, Carl Zeiss Vision GmbH, Aalen, Germany) at an accelerating voltage of 3.00 kV. Simultaneously, the surface and cross-sectional C and N elemental distributions of the membranes were scanned using SEM-EDS with an accelerating voltage of 15 kV for the energy spectrum mapping shot and SE2 secondary electron detectors. The surface wettability of the membranes was measured using a goniometer (SDC-100, Shengding Precision Shengding, Dongguan, China). The zeta potential of the membranes was measured using an electrodynamic analyzer (SurPass 3, AntonPaar, North Ryde, Australia) involving a 1 mM KCl solution with pH 3, 5, 7 and 9.

To characterize the fundamental properties of the membranes, the water uptake (WU), swelling ratio and ion exchange capacity (IEC) were determined. The weight and length of the sample in the wet state were obtained after the membrane had been soaked in deionized water for 12 h. After drying the wet samples in an oven at 50 °C for 12 h, the weight and length of the dried samples were measured and the average of the two measurements was taken as the final value. WU (%) was calculated using the wet and dry weights according to Equation (1):

(1) $$\mathrm{WU}\left(\%\right)=\frac{M_{wet}-M_{dty}}{M_{dry}}\times 100\%$$

where *M_wet_* and *M_dry_* mean the wet weight and dry weight (g) of the membrane sample, respectively.

The swelling ratio of the length (*SR_L_*, %) of the membrane was calculated using Equation (2):

(2) $$SR_{L}\left(\%\right)=\frac{L_{wet}-L_{dry}}{L_{dry}}\times 100\%$$

where *L_wet_* and *L_dry_* mean the wet and dry lengths (cm) of the membrane sample, respectively.

The membrane IEC was assessed using the classical acid–base titration method. Firstly, membrane sheets of known weight were immersed in 50 mL of 1 M HCl solution for 24 h. Subsequently, the membranes were thoroughly washed to remove any traces of hydrochloric acid (wash water was tested with AgNO_3_ to ensure no AgCl precipitation). Each sample was then immersed in 1 M NaCl solution for 24 h and then titrated with 0.10 M NaOH after the addition of two drops of phenolphthalein. The IEC (meq·g^−1^) of the membrane was calculated using Equation (3):

(3) $$\mathrm{IEC}\left(\mathrm{meq}\cdot g^{-1}\right)=\frac{V_{NaOH}\times C_{NaOH}}{M_{dry}}$$

where *C_NaOH_* (0.10 mol·L^−1^) is the concentration of NaOH and *V_NaOH_* (mL) is the volume of NaOH consumed during the titration.

The square resistance (SR, MΩ) of the membrane was measured using a four-probe conductivity meter (ST-2258C, Suzhou Jingle Electronics Co., Ltd., Suzhou, China) with a test membrane size of 2 cm × 2 cm, and the test was repeated 5 times for each membrane and the average value was taken.

### 2.4. Determination of Ionic Perm-Selectivity

A self-made DD cell was used to test the perm-selectivity of the membrane for ions. As shown in Figure 1b and Figure S1, two chambers each with a volume of 100 mL were separated by the membrane under study with an effective working membrane area of 9.08 cm^2^ and connected by ball mill mouth clamps. Both chambers were placed on a magnetic stirrer (IT-07 B3, Shanghai Yiheng Scientific Instrument Co., Ltd., Shanghai, China) at 400 rpm to ensure the homogeneity of the two solutions during the experiment. The volume of initial feed and receiving solution for each chamber was 100 mL. Samples were collected from the feed and receiver chambers separately at specific time intervals until Donnan equilibrium was reached.

First, the effect of different ion concentrations on the enrichment of NH_4_^+^ by DD was explored by configuring different concentrations of NH_4_Cl solutions and NaCl solutions (see Table S1 for details). Second, the effect of deposition substance and time on the selective permeability of the membrane to ions was investigated, and the specific experimental parameters were designed as shown in Table S2. Third, the effect of Mg^2+^ concentration on membrane permeability selectivity was investigated under the coexistence of NH_4_^+^/Mg^2+^. The experimental conditions are detailed in Table S3. Fourth, the permeability selectivity performance of different modified membranes for *C*[NH_4_^+^]:*C*[Mg^2+^] = 10:1 was investigated (see Table S2). Fifth, the stability of GO-PEI (20) and CEM membranes was tested for long-term operation under the conditions of 25 mM NH_4_Cl and 5 mM MgCl_2_ in the feed solution (50 mM NaCl in the receiving solution), respectively, for 8 consecutive cycles with a duration of 9 h each.

The concentration of NH_4_^+^-N in solution was tested by a UV spectrophotometer (Model 722, Shanghai Jingke Tianmei Scientific Instrument Co., Ltd., Shanghai, China) and Mg^2+^ by an atomic absorption spectrophotometer (TAS-990F, Beijing Pudian General Instrument Co., Ltd., Beijing, China). The ion removal rate was calculated by Equation (4):

(4) $$R=\frac{C_{0}-C_{f}^{t}}{C_{0}}$$

where R is the feed chamber ion removal rate, *C_0_* is the initial feed chamber ion concentration and $C_{f}^{t}$ is the feed chamber ion concentration at time *t*.

The ion flux through the membrane was calculated by Equation (5):

(5) $$J_{S}=\frac{C_{t}\times V_{t}-C_{0}\times V_{0}}{1000\times A\times t\times M_{S}}$$

where *J_S_* is the average ion molar flux (mol·m^−2^·h^−1^), *A* is the effective membrane area (m^2^), *C*_0_ is the initial ion concentration on the feed side (mg·L^−1^), *V*_0_ is the initial solution volume on the feed side (L), *C_t_* is the ion concentration after operation *t* (mg·L^−1^), *V_t_* is the solution volume after operation *t* (L) and *M_S_* is the relative molecular mass of the ion (g·mol^−1^).

The ion selectivity of the membrane was calculated from Equation (6):

(6) $$S=\frac{J_{NH_{4}^{+}}}{n\times J_{Mg^{2+}}}$$

where *S* is the selectivity of the NH_4_^+^/Mg^2+^, $J_{NH_{4}^{+}}$ is the flux of NH_4_^+^ (mol·m^−2^·h^−1^), $J_{Mg^{2+}}$ is the flux of Mg^2+^ (mol·m^−2^·h^−1^) and *n* is the molar concentration ratio of NH_4_^+^ to Mg^2+^.

## 3. Results and Discussion

### 3.1. Morphological and Physicochemical Characterizations of the Modified Membranes

**Figure 2:**
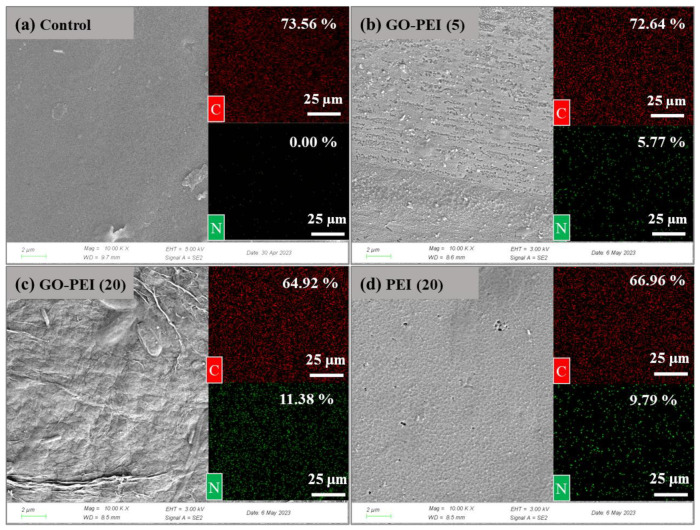
SEM and EDS scans of C (red) and N (green) elements images of (**a**) control membrane, (**b**) GO-PEI (5) membrane, (**c**) GO-PEI (20) membrane and (**d**) PEI (20) membrane surface.

The morphology and structure of an ion exchange membrane play an influential role in its electrochemical performance and durability [31]. As shown in Figure 2a, the surface of the control (unmodified) membrane is uniform. After GO-PEI deposition, the membrane surface became rough and showed some ridges (Figure 2b), which gradually shifted to distinct folds as the deposition time increased (Figure 2c). This is due to the disordered arrangement of the GO nanosheets becoming more pronounced as the stack thickness increases [32], favoring the agglomeration of the GO nanosheets as a result of PEI-GO binding. The surface of PEI (20) membrane is smoother than that of GO-PEI (20) membrane, partly because the presence of GO nanosheets leads to typical two-dimensional nanosheet stacking folds and the flexible polymer chains preferentially fill the concave areas [33], resulting in a less pronounced change in surface roughness. As can be seen from the cross-sectional images (see Figure S2), the GO-PEI (5) membrane, GO-PEI (20) membrane and PEI (20) membrane have surface coating thicknesses of ~35 nm, ~93 nm and ~70 nm, respectively, with firmly attached coatings on the membrane surface without obvious boundaries. Energy spectroscopy (EDS) analysis of the elemental composition and relative content changes on the membrane surface is shown in Table S4 and Figure 2. The N content of GO-PEI (5), GO-PEI (20) and PEI (20) membrane surfaces reached 5.77%, 11.38% and 9.79%, respectively, while no N-containing compounds were detected on the membrane surface of the control membrane, indicating that PEI successfully self-assembled on the membrane surface and the amount of attached PEI increased with increasing deposition time. According to the EDS analysis mapping of N, adding GO makes a denser and more homogeneous PEI layer on the membrane surface (see Figure 2c,d). The reason for this behavior is that the amine group on PEI can be nucleophilically substituted with the epoxy group on GO nanosheets to form covalent bonds [34], while the negatively charged GO nanosheets can attract the positively charged PEI [28] to be deposited on the membrane by electrostatic adsorption. The uniform distribution of N elements within the GO-PEI membrane and PEI membrane surface coating is further evidenced by the EDS analysis mapping of the membrane cross-section (see Figure S2), which shows a uniform deposition of GO and PEI on the membrane surface. Significantly, N elements were detected inside all the membranes after surface modification, indicating that during the impregnation process, some PEI molecules entered the membrane pores, which facilitated the enhancement of the connection between the coating and the base membrane. Successful deposition of PEI and GO on the membrane surface will enhance the membrane’s retention capacity of Mg^2+^, which is favorable for the selective permeation of NH_4_^+^ across the membrane.

**Figure 3:**
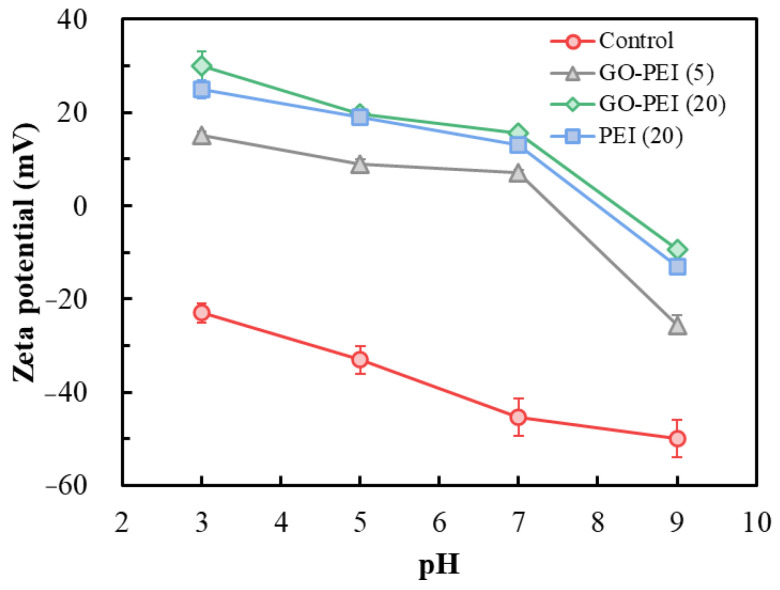
Zeta potential at the membrane surface.

Charge properties of the membrane surface were investigated using the zeta potential characterization (Figure 3). Because the pH of most ammonia nitrogen wastewater is in the neutral–slightly alkaline range [35], the most important region in Figure 3 is that for pH around 7. After impregnation of a GO-PEI mixture or PEI solution, the charge of the cation exchange membrane surface is changed from a negative to a positive value. The positively charged membrane surface has a different electrostatic repulsion for monovalent NH_4_^+^ and divalent Mg^2+^ (higher-valence cations are subject to stronger Coulombic forces [28]), thus facilitating the selective transport of NH_4_^+^ over Mg^2+^ by the membrane. The membrane potential can be ranked in order of its magnitude as: GO-PEI (5) < PEI (20) < GO-PEI (20). This indicated that the positive potential of the membrane surface gradually increased with the deposition of GO-PEI. Meanwhile, the addition of GO is more favorable for the deposition of PEI, so the potential of GO-PEI (20) is slightly higher than that of PEI (20) with the same deposition time of 20 min, that is, the addition of GO is beneficial for the enhancement of membrane NH_4_^+^ selectivity.

The change in wettability and hydrophilicity of the membrane was assessed by measuring the water contact angle (CA) and water uptake (WU) of the membrane (Table 1). GO-PEI or PEI loading reduces the CA of the membrane. Also, the CA of the GO-PEI membrane can be reduced from 67.10° to 57.20° with increasing deposition time. Additionally, the CA of the GO-PEI (20) membrane (57.20°) is smaller than that of the PEI (20) membrane (65.50°) with the same deposition time, indicating that the addition of GO is more favorable for increasing the hydrophilicity of the membrane. The results for WU were consistent with the CA, further demonstrating that the GO-PEI (20) membrane provides a greater hydrophilicity and is therefore more suitable for DD applications. The increase in hydrophilicity is because PEI molecules contain many hydrophilic -NH_2_ groups. The number of amino groups introduced to the membrane by PEI increases with the deposition time, and therefore the hydrophilicity of the surface is further enhanced [32]. On the other hand, due to the hydrophilic nature of the GO nanosheet, which is rich in a large amount of polar oxygen-containing groups such as -OH, C-O-C and -COOH, the composite membrane becomes significantly more hydrophilic [36]. GO-PEI (20) membrane has a higher WU of 21.07%, but its swelling rate (*SR_L_*) is smaller than that of the control membrane by approximately 2.22%, which indicates a better stability of GO-PEI (20) membrane under humid conditions. The reduction in *SR_L_* is due to the cross-linking of PEI, both on the membrane surface and its interior, as shown in Figure S2. The lower swelling rate predicted that the GO-PEI (20) membrane could better maintain the efficient and selective separation of NH_4_^+^/Mg^2+^ in a long-term operation.

The IEC results show that utilizing GO and PEI on the CEM led to changes in ion exchange capacity of the membrane (Table 2). GO-PEI (20) membrane showed an IEC of 1.61 meq·g^−1^, which is lower than that of the control membrane. Probably, this is because during the modification process a small amount of PEI molecules entered into the interior of the CEM [32] and occupied some ion exchange sites [37], resulting in a slightly reduced ion exchange capacity of the modified membrane. In any case, the reduction is small and the IEC is comparable with the IEC value region of 1.5 to 1.8 meq·g^−1^ for Neosepta, the CMX provided by the manufacturer (Astom Corp., Yamaguchi, Japan). Therefore, the modification of the membrane surface by GO-PEI will not significantly reduce the NH_4_^+^ transport rate across the membrane. The GO-PEI (20) membrane has an SR test result of 0.328 MΩ (Table 2), which is slightly lower than that of the other modified membranes but is comparable to that of the control membrane.

### 3.2. Effect of Donnan Dialysis Operating Parameters

**Figure 4:**
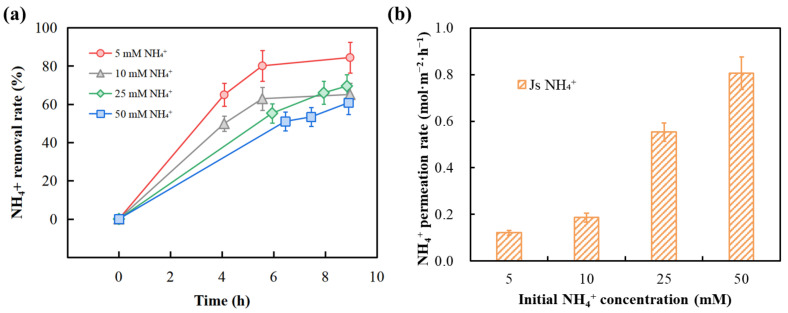
Variation of (**a**) NH_4_^+^ removal rate with time for different ion concentrations (the initial molar Na^+^ concentration is twice the initial concentration of NH_4_^+^), (**b**) NH_4_^+^ average permeation rate.

The optimum concentrations of NH_4_^+^ (target counter ion) and Na^+^ (driving counter ion) for the enrichment of ammonia nitrogen by DD were investigated. Based on our previous work, the NH_4_^+^ to Na^+^ concentration ratio was fixed at 1:2 toward an efficient concentration-driven performance [15]. Figure 4a shows that the NH_4_^+^ removal (calculated by Equation (4)) decreases with increasing NH_4_^+^ concentration in the feed solution. The average fluxes of NH_4_^+^ in the DD system with initial feed solution NH_4_^+^ concentrations of 5, 10, 25, and 50 mM were 0.121, 0.186, 0.553 and 0.807 mol·m^−2^·h^−1^ (Figure 4b), respectively, which was opposite to the change in NH_4_^+^ removal rates. Although the increase in NH_4_^+^ concentration in the feed solution increased the NH_4_^+^ mass transport rate ($C_{0}-C_{f}^{t}$) to some extent, this increase was not significant compared to the increase in the NH_4_^+^ initial concentration ($C_{0}$) [11]. Donnan equilibrium was almost reached at around 9 h under lower concentrations, which may require more time in case of higher concentrations. As expected, an increase in NH_4_^+^ transport rate was more pronounced when the NH_4_^+^ concentration in the feed solution was increased from 10 to 25 mM. Considering the cost of reagents, time and experimental effectiveness, the use of a feed NH_4_^+^ concentration of 25 mM and an average run time of 9 h were selected to optimize the DD process performance in the experimental unit used.

### 3.3. Counter Ion Transport Behavior and Selectivity

**Figure 5:**
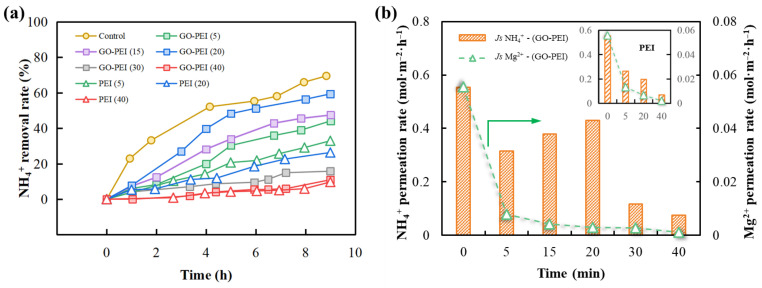
Separation effect of different modified membranes on pure ammonium and pure magnesium salts, (**a**) variation of NH_4_^+^ removal rate in the feed chamber with time, (**b**) variation of the NH_4_^+^ and Mg^2+^ average permeation rate with deposition time (the inset figures show the test results of PEI-modified membranes). The feed solution is a 25 mM NH_4_Cl/2.5 mM MgCl_2_ solution and the receiving solution is a 50 mM NaCl solution.

The variations of NH_4_^+^ removal rate for the control membrane and for the PEI-modified membrane and GO-PEI-modified membrane with deposition time are shown in Figure 5a for a feed solution with 25 mM NH_4_Cl and a receiving solution with 50 mM NaCl. Both GO-PEI and PEI depositions resulted in a decrease in the NH_4_^+^ removal rate, with the lowest decrease (~10%) occurring with the GO-PEI (20) membranes. This decrease can be attributed to the positively charged PEI layer limiting the rate of positively charged NH_4_^+^ ions crossing the membrane. As shown in Figure 5b, for the PEI-modified membrane, the permeation rates of the membrane to NH_4_^+^ and Mg^2+^ decreased from 0.553 and 0.055 mol·m^−2^·h^−1^ to 0.071 and 0.002 mol·m^−2^·h^−1^, respectively, with increasing deposition time. It is important to note that due to the addition of GO nanosheets, the permeability of the GO-PEI (20) membrane to NH_4_^+^ was increased by 1.17 times compared to that of the PEI (20) membrane, while the permeability to Mg^2+^ was only 57.13% of that of PEI (20) membrane. This is because the negatively charged GO nanosheets can attract the positively charged NH_4_^+^ to transport into the membrane but adsorb the metal cations (Mg^2+^), preventing them from entering the membrane. In addition, the presence of GO allows more PEI to be deposited on the membrane surface, which to a certain extent increases the positive charge on the membrane surface, leading to a stronger electrostatic repulsion of Mg^2+^, which in turn leads to a further decrease in Mg^2+^ permeability. Nevertheless, the permeability of GO-PEI (40) membranes to NH_4_^+^ decreased sharply from 0.429 mol·m^−2^·h^−1^ to 0.074 mol·m^−2^·h^−1^ when the deposition time continued to be extended. This can be attributed to a reduction in membrane surface defects and a significant increase in ion permeation resistance as a result of longer deposition times [37]. Thus, impregnation in the GO-PEI mixture for 20 min was the optimum condition for the subsequent study.

**Figure 6:**
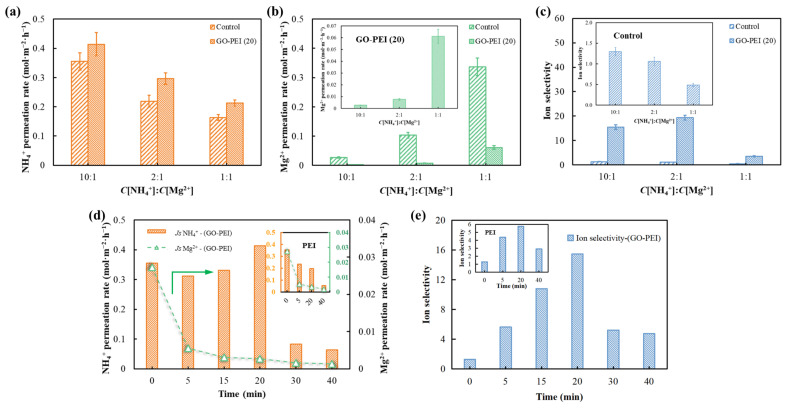
Effect of co-presence of different concentrations of Mg^2+^ on the separation effect of base membrane and GO-PEI (20) membrane (*C*[NH_4_Cl] = 25 mM, *C*[NaCl] = 50 mM and *C*[MgCl_2_] = 2.5–25 mM). (**a**) NH_4_^+^ permeation rate, (**b**) Mg^2+^ permeation rate and (**c**) ion selectivity. (**d**) NH_4_^+^, Mg^2+^ permeation rates and (**e**) ion selectivity when membranes made with different deposition times were used to treat mixed salt solutions with *C*[NH_4_^+^]:*C*[Mg^2+^] = 10:1 (the inset figures show the test results of (**b**) GO-PEI (20) membrane, (**c**) control membrane, (**d**) PEI membrane and (**e**) PEI membrane, respectively, under the corresponding conditions).

GO-PEI (20) membrane was used to investigate the effect of Mg^2+^ concentration of mixed salt solutions on the separation performance of the membrane, where the initial NH_4_Cl concentration in the feed solution was fixed at 25 mM and the receiving solution contained 50 mM NaCl. As shown in Figure 6a,b, both the control membrane and the GO-PEI (20) membrane showed a decrease in NH_4_^+^ permeation rate and increase in Mg^2+^ permeation rate with increasing Mg^2+^ concentration. Different from the ammonium and magnesium salt pure solution test (Figure 5b), there was competition between the two cations in the mixed salt solution, resulting in a decrease in both NH_4_^+^ and Mg^2+^ permeation rates [14]. In Figure 6b,c, it can be seen that the Mg^2+^ permeation rate of the control membrane overcomes the NH_4_^+^ permeation rate when *C*[NH_4_^+^]:*C*[Mg^2+^] = 1:1. This indicates that when the Mg^2+^ concentration of the solution is excessive, the anions in the solution will concentrate around the positive charge sites on the membrane surface, weakening its electrostatic repulsion of the cations in solution. That is, a charge shielding effect occurs, which increases the Mg^2+^ permeation rate and reduces the membrane’s selectivity for monovalent/divalent ions [38].

Under the same Mg^2+^ concentration conditions, as shown in Figure 6a,b, the NH_4_^+^ permeation rate was greater and the Mg^2+^ permeation rate was smaller for the GO-PEI (20) membrane compared to the control membrane. Moreover, the Mg^2+^ permeation rate at *C*[NH_4_^+^]:*C*[Mg^2+^] = 10:1 was close to 0 (0.0026 mol·m^−2^·h^−1^). The ion selectivity can be assessed by calculating the ion permeability ratio of NH_4_^+^ to Mg^2+^ (Figure 6c). When *C*[NH_4_^+^]:*C*[Mg^2+^] = 2:1, the ion selectivity of the GO-PEI (20) membrane reaches a maximum of 19.47, which is much greater than the ion selectivity of the control membrane (1.06). When the Mg^2+^ concentration increased to the same level as NH_4_^+^, the ion selectivity of the GO-PEI (20) membrane and the control membrane decreased from 19.47 and 1.06 to 3.48 and 0.48. This suggests that the charge repulsion effect plays an important role in the ion exchange process, with divalent Mg^2+^ being repelled by the high density of positive charges on the surface of the GO-PEI (20) membrane when the magnesium salt concentration is low, thus exhibiting lower Mg^2+^ permeability and higher ion selectivity.

Since *C*[NH_4_^+^]:*C*[Mg^2+^] ranges from 13:1 to 5:1 in low- to medium-concentration ammonia nitrogen wastewater, a mixed salt solution with *C*[NH_4_^+^]:*C*[Mg^2+^] = 10:1 was chosen for the comparative study of ion permeation rate and selectivity of different membranes in order to better mimic the actual wastewater. The ion separation performance of the membranes during the separation of mixed salt solutions is shown in Figure 6d. For the GO-PEI membrane, as the deposition time increased, the NH_4_^+^ permeation rate increased and then decreased and the Mg^2+^ permeation rate continued to decrease. The best separation effect was achieved when the GO-PEI membrane was deposited for 20 min, with an NH_4_^+^ permeation rate of 0.414 mol·m^−2^·h^−1^, which was greater than that of the control membrane, and the Mg^2+^ permeation rate decreased to 0.0027 mol·m^−2^·h^−1^. For PEI membranes, the permeability of NH_4_^+^ and Mg^2+^ showed a continuous decreasing trend with increasing deposition time. Meanwhile, for the same deposition time, the NH_4_^+^ permeation rate is ranked as: PEI membrane < GO-PEI membrane and the Mg^2+^ permeation rate is ranked as: PEI membrane > GO-PEI membrane. This further indicates that the addition of GO increases the NH_4_^+^ permeation rate and decreases the Mg^2+^ permeation rate. The ion selectivity of the modified membranes is shown in Figure 6e, which shows that the ion selectivity of GO-PEI membranes increases and then decreases with increasing deposition time. The ion selectivity of the PEI (20) membrane was only 5.74, which was lower than that of GO-PEI (20), further indicating that GO plays an important role in the membrane separation process.

**Figure 7:**
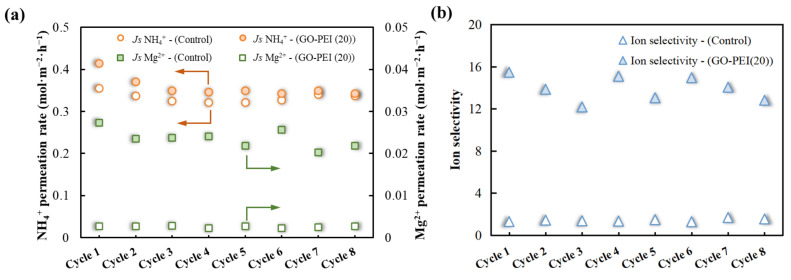
(**a**) NH_4_^+^ and Mg^2+^ permeation rate and (**b**) ion selectivity for consecutive batch cycles of [NH_4_^+^]:[Mg^2+^] = 10:1 mixed salt solutions with control and GO-PEI (20) membranes (*C*[NH_4_Cl] = 25 mM, *C*[NaCl] = 50 mM and *C*[MgCl_2_] = 2.5 mM).

A long-term stable operation is an important indicator for evaluating membrane performance. Therefore, dedicated experiments were performed to assess the operation stability, by performing eight sequential batches, each one for 9 h, for a total of 72 h. As shown in Figure 7a, the NH_4_^+^ and Mg^2+^ permeabilities of both the control and GO-PEI (20) membranes decreased slightly over time, but overall, the GO-PEI (20) membrane had slightly higher NH_4_^+^ permeation than the control membrane. Over time, the Mg^2+^ permeation rate of GO-PEI (20) membrane remained around 0.0026 mol·m^−2^·h^−1^, which is lower than the 0.024 mol·m^−2^·h^−1^ of the control membrane. As shown in Figure 7b, the ion selectivity of the GO-PEI membrane for NH_4_^+^/Mg^2+^ remained around 14.53 after 72 h of testing, which was much higher than that of the control membrane (~1.36). Thus, the results obtained confirm the potential of GO-PEI membranes prepared using the self-assembly method for the efficient separation of NH_4_^+^.

### 3.4. Mechanism of NH_4_^+^ Ion-Selective Transport in GO-PEI Membranes

**Figure 8:**
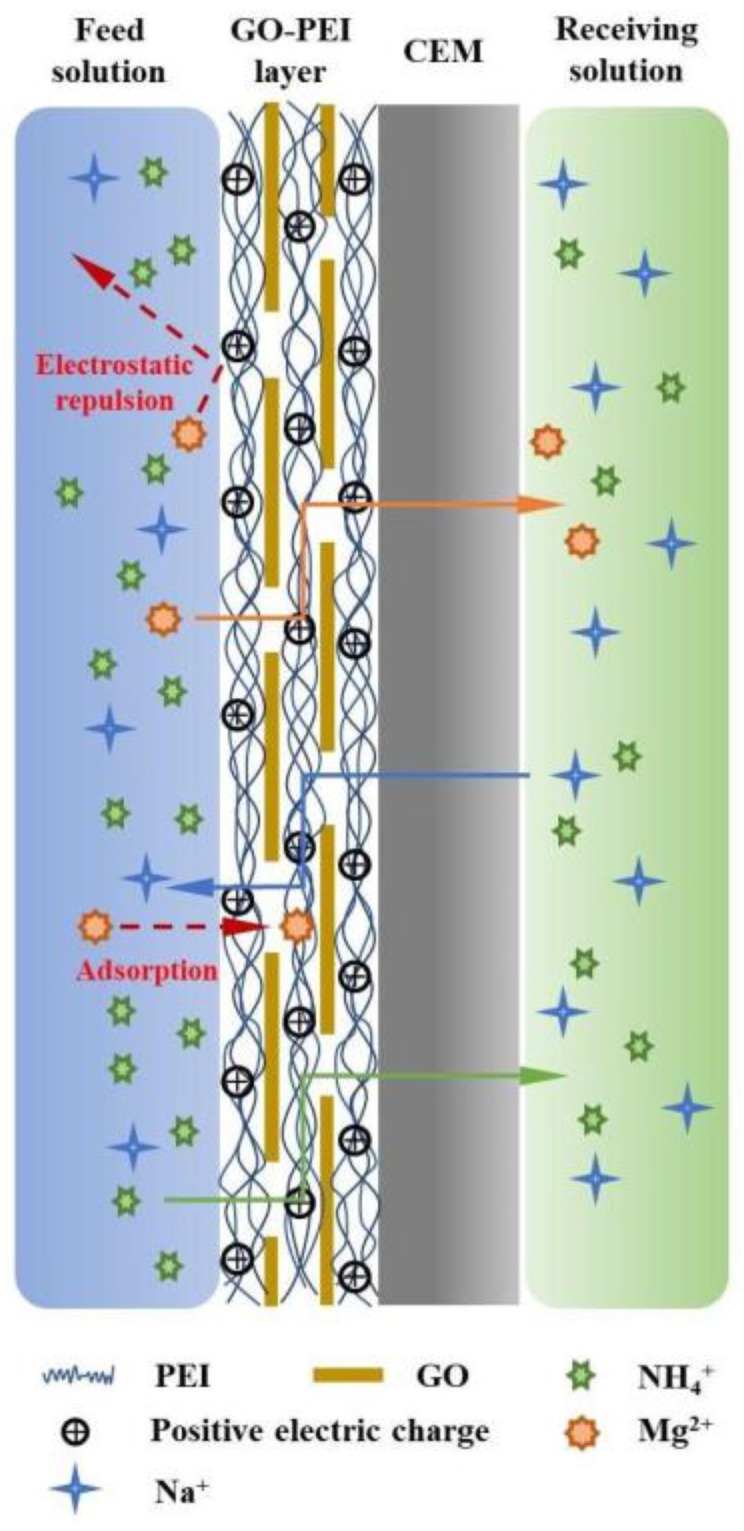
Schematic diagram of the GO-PEI membrane separation process.

A schematic drawing of the GO-PEI membrane NH_4_^+^/Mg^2+^ DD separation is presented in Figure 8. The -NH_2_ in the positively charged PEI can be covalently cross-linked with the -COOH at the edge of the negatively charged GO during the impregnation process, forming a GO-PEI layer with controlled surface charge by layer self-assembly at the CEM [39]. The GO-PEI-modified layer changes the charge on the membrane surface from negative to positive (see Figure 3), granting stronger electrostatic membrane surface repulsion of highly valent cations (e.g., Ca^2+^, Mg^2+^) [40]. Compared to PEI membrane without GO modification, the GO-PEI membrane has a higher positive surface charge because the negatively charged GO induces more positively charged PEI to load on the membrane surface at the same deposition time (see Figure 2). In addition, GO can form π–metal cation conjugation with Mg^2+^ and adsorb part of Mg^2+^ [28], thus achieving selective retention of Mg^2+^. Wang et al. [39] prepared ion-selective separation membranes using GO-PEI deposited on the surface of microfiltration membranes, performing monovalent/multivalent ion-selective separation through two-dimensional nanochannels with electrostatic repulsion and size-screening capabilities. Different from the report in the literature, the TEM observation of the sample sections (see Figure S3) revealed that the GO nanosheets were not uniformly stacked on the CEM surface to form two-dimensional nanochannels. This is because positively charged PEI combined with monolayer GO will change the surface potential of GO nanosheets, leading to the agglomeration of GO nanosheets during spontaneous deposition [41]. Hence, in this study, GO was unable to achieve selective screening of NH_4_^+^/Mg^2+^ through the construction of nanochannels. Part of the PEI molecules penetrated inside the CEM (see Figure S2), which made the PEI layer adhere more firmly to the CEM, and the positively charged PEI inside the membrane pores could further enhance the rejection of Mg^2+^ by the CEM. In summary, the presence of GO facilitated the loading of PEI on the CEM membrane, and the selective adsorption of Mg^2+^ by GO and the stronger electrostatic repulsion of Mg^2+^ by PEI resulted in an efficient separation of NH_4_^+^ and Mg^2+^ by the GO-PEI membrane.

## 4. Conclusions

Based on the membrane material requirements in the process of ammonia nitrogen recovery from wastewater by DD technology, in this work, a GO-PEI membrane was prepared for the selective separation of NH_4_^+^/Mg^2+^ by dip-coating on the self-assembled CEM surface to form a GO-PEI-modified layer. The deionization of the amine group of PEI leads to high-density positive charges on the membrane surface, thereby creating enhanced electrostatic repulsion for divalent cations (Mg^2+^) to improve the selectivity of NH_4_^+^/Mg^2+^. GO nanosheets induce more PEI molecules to be grafted on the CEM surface, enhance the positive charge of the membrane surface and adsorb part of the Mg^2+^ by π–metal cation conjugation, further enhancing the selectivity of the membrane. The ideal mass transport rate (single ion) of the GO-PEI (20) membrane for NH_4_^+^ was equal to 0.429 mol·m^−2^·h^−1^, which was close to that of the unmodified membrane (0.553 mol·m^−2^·h^−1^), whereas the ideal mass transport rate for Mg^2+^ was 0.003 mol·m^−2^·h^−1^, being only 5.36% of the ideal mass transport rate for Mg^2+^ of the unmodified membrane (0.056 mol·m^−2^·h^−1^). The NH_4_^+^/Mg^2+^ selectivity ratio for the mixed salt solution test was 15.46, which was much greater than that for the unmodified membrane (1.36) and remained stable in several consecutive batches of DD tests, thus showing the potential of using the modified membrane for an NH_4_^+^-N-selective recovery.

## Figures and Tables

**Table 1:** Membrane wettability parameters.

Membrane Type	Contact Angle (°)	Water Uptake (%)	Swelling Rate (%)
Control	74.90 ± 1.50	12.71 ± 0.01	5.19 ± 0.02
GO-PEI (5)	67.10 ± 0.80	15.29 ± 0.01	4.59 ± 0.02
GO-PEI (20)	57.20 ± 0.60	21.07 ± 0.01	2.22 ± 0.01
PEI (20)	65.50 ± 0.70	17.31 ± 0.01	2.64 ± 0.01

**Table 2:** The IEC and SR for the prepared membranes.

Membrane Type	IEC (meq · g^−1^)	SR (MΩ)
Control	1.85	0.349
GO-PEI (5)	1.72	0.339
GO-PEI (20)	1.61	0.328
PEI (20)	1.68	0.364

## Data Availability

Not applicable.

## Supplementary Materials

The following supporting information can be downloaded at: https://www.mdpi.com/article/10.3390/membranes13080726/s1, Figure S1: Photograph of the Donnan dialysis unit used, Figure S2: SEM and EDS scans of C (red) and N (green) elements images of (a) control membrane, (b) GO-PEI (5) membrane, (c) GO-PEI (20) membrane and (d) PEI (20) membrane cross-section, Figure S3: A TEM image of a cross-sectional section of the GO-PEI (20) membrane, Table S1: Design of ionic concentration investigations, Table S2: Design of deposition conditions, Table S3: Exploratory design of the effect of Mg^2+^ concentration, Table S4: EDS scan data for membrane surfaces and cross-sections.
