# Supplementary material for: Graphene Oxide/Polyethyleneimine-Modified Cation Exchange Membrane for Efficient Selective Recovery of Ammonia Nitrogen from Wastewater
Source: Membranes (Basel). 2023 Aug 10;13(8):726. doi: 10.3390/membranes13080726 (PMC10456636; doi:10.3390/membranes13080726)
Supplement: Supplementary file 1 [file membranes-13-00726-s001.zip › membranes-2534064-supplementary.pdf]

Supporting Information

Graphene oxide / polyethyleneimine modified cation exchange membrane for efficient selective recovery of ammonia nitrogen from wastewater

Yuanyuan Yu[a], Qin Zeng[a], Haoquan Zhang[a], Maoqin Ao[a], Jingmei Yao[a], Chun Yang[a], Svetlozar Velizarov[b]*, Le Han[a]*

[a] *Key Laboratory of the Three Gorges Reservoir Region's Eco-Environment, Ministry of Education, College of Environment and Ecology, Chongqing University, Chongqing 400045, China.*

[b] *LAQV / REQUIMTE, Department of Chemistry, NOVA School of Science and Technology, FCT NOVA, Universidade NOVA de Lisboa, 2829-516 Caparica, Portugal.*

***Corresponding authors:** Svetlozar Velizarov, Universidade NOVA de Lisboa, Portugal; Le Han, Chongqing University, China.

E-mail addresses: s.velizarov@fct.unl.pt (S. Velizarov)**;** lehan@cqu.edu.cn (L. Han)

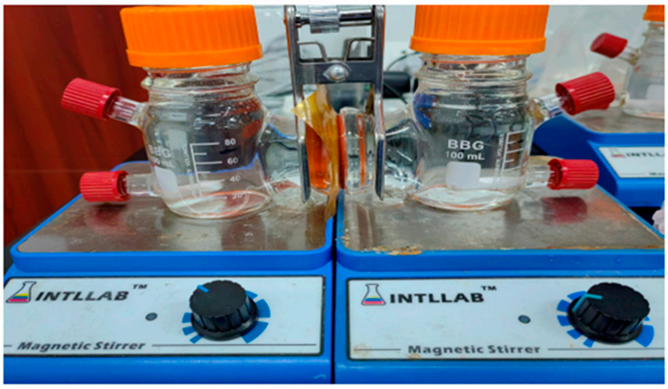

Figure S1. Photograph of the Donnan dialysis unit used.

Table S1. Design of ionic concentration investigations

Liquid[a] concentration (mM)		Membrane type	Sampling interval time (h)	Running times (h)
NH_4Cl	NaCl			
5	10	CEM	1	9
10	20			
25	50			
50	100			

[a] NH_4Cl is the feed solution and NaCl is the receiving solution.

Table S2. Design of of deposition conditions

Deposition conditions		Membrane type	Liquid type		Sampling interval time (h)	Running time (h)
Time (min)	Material		Feed	Receiving		
0						
5						
15	GO-PEI					
20			25 mM NH_4Cl			
30			or	50 mM NaCl		
40			2.5 mM $MgCl_2$			
5						
20	PEI					
40		CEM			1	9
0						
5						
15	GO-PEI					
20			25 mM NH_4Cl			
30			and	50 mM NaCl		
40			2.5 mM $MgCl_2$			
5						
20	PEI					
40						

Table S3. Exploratory design of the effect of Mg^{2+} concentration

Liquid concentration (mM)			$C[NH_4^+] : C[Mg^{2+}]$	Membrane type	Sampling interval time (h)	Running time (h)
Feed		Receiving				
NH_4Cl	$MgCl_2$	NaCl				
25	2.5	50	10:1	GO-PEI membrane/ PEI membrane/ CEM	1	9
25	12.5	50	2:1			
25	25	50	1:1			

Table S4. EDS scan data for membrane surfaces and cross-sections

Membrane type	Elemental content (%)							
	Surface				Cross-section			
	C	O	S	N	C	O	S	N
Control	73.56	17.88	7.39	0.00	68.97	19.99	3.91	1.81
GO-PEI (5)	72.64	10.11	6.87	5.77	67.13	10.66	2.34	12.31
GO-PEI (20)	64.92	16.37	4.11	11.38	60.99	12.97	1.98	20.68
PEI (20)	66.96	14.02	5.08	9.79	63.91	16.46	3.60	12.30

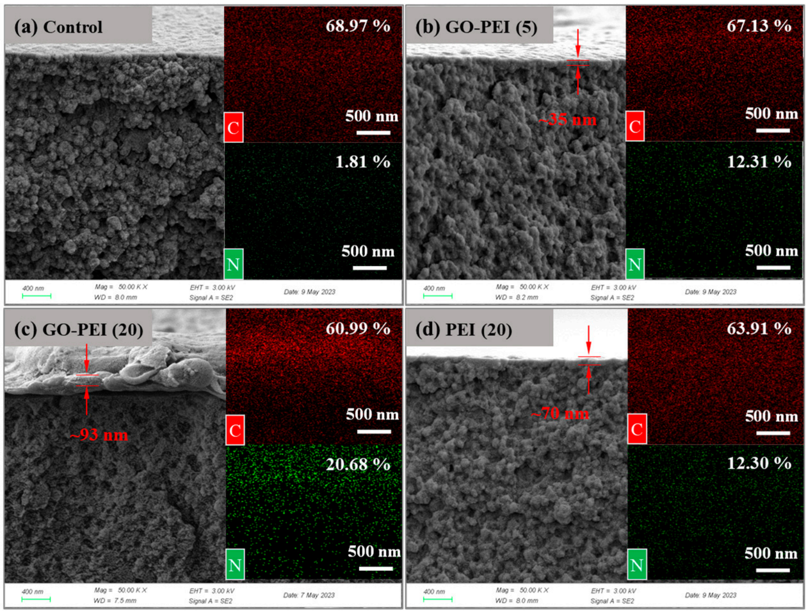

Figure S2. SEM and EDS scans of C (red) and N (green) elements images of (a) control membrane, (b) GO-PEI (5) membrane, (c) GO-PEI (20) membrane and (d) PEI (20) membrane cross-section.

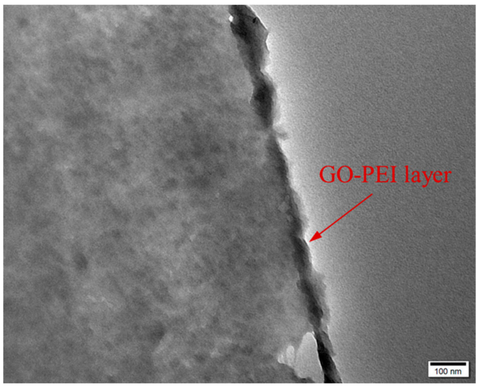

Figure S3. A TEM image of a cross-sectional section of the GO-PEI (20) membrane.
